# Evaluation of Greek Cattle Carcass Characteristics (Carcass Weight and Age of Slaughter) Based on SEUROP Classification System

**DOI:** 10.3390/foods9121764

**Published:** 2020-11-28

**Authors:** Kostoula Nikolaou, Panagiota Koutsouli, Iosif Bizelis

**Affiliations:** 1 Department of Bovine Sector and Equity, Directorate General of Agriculture, Directorate of Animal Husbandry Systems, Hellenic Ministry of Rural Development and Food, Veranzerou 46, 10176 Athens, Greece; knikolaou@minagric.gr; 2 Laboratory of Animal Breeding and Husbandry, Department of Animal Science, Faculty of Animal Biosciences, Agricultural University of Athens, 11855 Athens, Greece; jmpiz@aua.gr

**Keywords:** beef, local breeds, carcass weight, age of slaughter, SEUROP system

## Abstract

In Greece, all cattle carcasses produced from a variety of breed types are classified according to the SEUROP system. The objective of this study was to evaluate Greek carcass characteristics such as carcass weight and age of slaughter based on SEUROP classification system (muscle conformation and fat deposit classes) and to describe the effect of main factors such as breed, gender, year of slaughter, farm’s geographical region and month of slaughter on these carcass parameters. It is the first study that evaluates local breeds, revealing the wide diversity of the Greek cattle breeding conditions. The analyzed records consisted of 323,046 carcasses from 2011 to 2017. All the examined factors significantly affected the mean carcass weight (298.9 ± 0.2 kg) and the mean slaughter age (559.1 ± 0.3 days). Carcasses from beef meat breeds had on average higher mean carcass weight while the local breeds had lower. The mean slaughter age and carcass weight were higher in winter than in summer. The local and the dairy breeds were classified in similar muscle conformation classes. Finally, Greek cattle carcasses from almost all regions were satisfactory for their quality carcass traits with good muscle conformation (R, O and U class) and low-fat deposit (class 1 to 3).

## 1. Introduction

Beef is the third most widely consumed meat in the world and is considered to be a highly nutritious and valued food [1]. Intended for culinary and meat-processing purposes beef meat must meet certain qualitative requirements in terms of its sensory characteristics such as a suitable color, a desirable flavor features, an appropriate texture and a high level of tenderness [2]. The characteristics of beef carcass have a significant effect on meat quality and play a decisive role in determining its value. The value of the animal carcass and the cost of producing that carcass determine the profitability in cattle production systems [3]. Furthermore, major factors that affect the value of the carcasses and the cost of meat production include the animal’s genotype, nutritional and management practices applied on the farm. The quality classification systems of carcasses are widely used as tool in the beef industry, making the business transactions easier while at the same time support the primary sector by providing it with useful information. The term “classification” defined as a set of descriptive terms describes the features of the carcass, which are useful to those involved in the trading of carcasses [4]. In the European Union (EU), the adoption of the SEUROP classification system within the member states established in 1981 is obligatory to record, monitor and collect data according to EU legislation [5] that concerns the carcass weight, the gender and the age of slaughter, the muscular conformation and the state of fattening of the carcass. Therefore, these data are measurable indicators that determine the quality characteristics and define the economic value of the carcass.

The general view by all sectors involved (slaughterhouses, producers, services) for the beef carcass classification system in the EU is that it operates well and provides, mainly for producers, a reliable basis for the deadweight sale of finished cattle [6]. Although in a recent study [7] is considered that, the SEUROP grid may be based on global indicators but it does not consider the carcass as a complex and heterogeneous entity, so, in the same SEUROP classification, it could include different muscles with higher or lower commercial value. The lack of a strong and clear link between sensory scores and European carcass classification standards shows that the European beef industry can not only rely on them but also needs to integrate quality into the carcass value [8]. Therefore, a study proposed alternative measures to be included in the SEUROP system in order to enable meat quality and to deliver consistent beef quality to the consumers [8].

While the SEUROP carcass grading system can be appropriate today, the ongoing changes in the production and marketing of cattle internationally could require in the future providing additional meat quality characteristics. A significant number of research studies tried to investigate the relationship between meat quality characteristics and the carcass parameters of SEUROP system mainly muscle conformation and fat deposit. Regarding marbling, a recent study [9] indicated that European classification scores explain only a slight proportion of the variance in marbling score (32%, 46%, 34% and 21% for the entire cattle group, young bulls, females and steers, respectively). Moreover, a significant correlation was observed among carcass yield and SEUROP conformation and fatness scores with intramuscular fat, slaughter body weight and hot carcass weight [10]. As a first step in developing a new way to assess the overall quality of beef carcasses in Europe it was proposed [11] a set of 5 indicators to include in the SEUROP system [hind quarter weight, meat color, retail-cut yield, rib-eye area and marbling score].

Beef production in the EU is approximately stable around 600,000 tons per month and holds the 3rd position after the United States of America (USA) and the Federative Republic of Brazil. In 2018, almost 7930 million tons of bovine meat (calve, young cattle, heifer, cow, bull and bullock) were produced in the EU from 87 million bovine animals. The highest production of European beef meat came from France (19%), Germany (15%) and the United Kingdom (12%), while almost half of the veal production in EU came from Spain (23%) and the Netherlands (23%) [12]. Considering that, the primary production of beef in EU consists of almost two thirds of dairy cows it is obvious that milk production is the main objective for most European cattle farms and only a small part of their income comes from beef production. 

The average carcass weight in EU increased by about 24 kg/head from 2000 to 2015 [13], despite the fact that the EU beef consumption corresponds to 10.9 kg/capita/year with large fluctuations between its member states [14].However, beef consumption in the developed world has been declining for the past 20 years, with rates falling to 12% in the EU, 19% in the USA and 20% in Australia [15]. Many studies have evaluated the causes of this declining trend that could be attributed to the negative criticism received by beef meat on issues related to the environment, public health, safety and authenticity, including the lack of consistency in the quality of beef meat [15,16,17,18]. Since 2003, the World Health Organization (WHO) has developed specific guidelines that pointed out the relationship between dietary fat and incidence of lifestyle diseases [19]. Supporting not only sensory and nutritional quality is therefore a priority issue for the beef meat industry in order to overcome the decline trend in consumption [7]. However, consumers increasingly appear to prefer high-quality meat cuts that are, characterized by consistently high levels of eating quality [18].

Numerous studies evaluating the endogenous factors that affect the quality characteristics of carcasses, pointed out the effect of genotype and gender of the cattle [20,21,22,23,24,25,26,27,28]. Regarding the exogenous factors, regional differences due to climate and geographical morphology heterogeneity, affect the calving season, weaning weight, reproductive efficiency, feed costs and the animal’s growth gain, configuring the final quantity and quality characteristics of the slaughtered cattle [29,30].

The beef sector in Greece has a great interest for study because it presents a lot of peculiarities. Being the southernmost country in Europe, it differs significantly, not only for the climatic conditions in contrast to the northern European countries, but also for the diversity in the breeding conditions of bovine animals. In addition, there is a large variety of cattle breeds that are bred and slaughtered in Greece, because local breeds do not meet the Greek beef meat’s demand. Specifically, the Greek local breeds have not evaluated in the past according their carcass characteristics either compared with other European beef breeds.

This study aims:(i) to describe the effect of main factors (breed, gender, year of slaughter, farm’s geographical region and month of slaughter) on the carcass weight and age of slaughter for various types of cattle carcasses (calve, young cattle, heifer and young bull); (ii) to evaluate the beef carcasses produced in Greece based on the SEUROP classification system. It is hypothesized that the information concerning the effect of the farm’s geographical region on the carcass characteristics will give more insight on the development of the sector. In addition, for the first time it will be presented the carcass characteristics from four Greek meat breed carcasses (Greek Red, Greek Blonde, Vrachiceratiki or Greek Brachyceros and Local cattle) as well as from the Greek Buffalo (Bubalus bubalis).

## 2. Materials and Methods

Field data (n = 979,806) were collected from the Integrated Veterinary Information System (IVIS) and the online application “ARTEMIS” of the Hellenic Agricultural Organization “ELGO-DIMITRA” from 132 approved slaughterhouses, geographically distributed in all 13 regions of the country from years 2011 to 2017. The registration of the data is obligatory based on the national legislation. The data included the gender, the breed and the geographical region of the farm, the date of birth and the date of slaughter, the carcass weight and the SEUROP classification categories.

The EU definition of carcass is ‘’the whole body of a slaughtered animal as presented after bleeding, evisceration and skinning’’. According to European legislation [31], the beef carcass is weighed as soon as possible after slaughter and not later than 60 min after the animal has been stuck and the presentation of the beef carcass should be (a) without the head and the feet; the head shall be separated from the carcass at the atloido-occipital joint and the feet shall be severed at the carpametacarpal or tarsometatarsal joints; (b) without the organs contained in the thoracic and abdominal cavities with or without the kidneys, the kidney fat and the pelvic fat; (c)without the sexual organs and the attached muscles and without the udder or the mammary fat.

The EU classification system classified bovine carcasses according to their gender and age into 6 categories using the letters A, B, C, D, E and Z. The definition of each letter is A: carcasses of uncastrated male animals aged from 12 months to less than 24 months; B: carcasses of uncastrated male animals aged from 24 months; C: carcasses of castrated male animals aged from 12 months; D: carcasses of female animals that have calved; E: carcasses of other female animals aged from 12 months and Z: carcasses of animals aged from eight months to less than 12 months. In addition to the latter categories, in European legislation [31] there is one more with the letter V for the carcasses of animals aged less than eight months. The beef carcasses in category V were not obliged to be classified according to SEUROP system. In our study, the category C was not used because there were no carcasses slaughtered in Greece in this category.

The SEUROP system defines six classes in order to classify carcasses according to their muscle conformation. The S class is ‘’superior’’; the E class is ‘’excellent’’; the U class is ‘’very good’’; the R class is ‘’good’’; the O is ‘’fair’’ and the P class is ‘’poor’’. Regarding to the fat deposit, the EU system classified bovine carcasses into five classes using the numbers 1–5. Specifically, class 1 is low; class 2 is slight; class 3 is average; class 4 is high; class 5 is very high.

The final selected dataset for analysis excluded the crossbred animals and consisted from 323,046 carcasses derived from 24 purebred cattle breeds including all animals with age of slaughter from 210 to 975 days with a sufficient number of observations (>100).

For the statistical processing of carcass weight and age of slaughter data, the analysis of variance was used (one-way ANOVA) in order to detect significant differences between the relative means for breed, gender, slaughter year, slaughter month, farm’s geographical region and categories of carcass classification, muscle conformation and fattening. For multiple comparisons, the Bonferroni criterion used was set at significance level of *p* ≤ 0.05. All statistical analyses were performed with the statistical program SPSS Statistics for Windows (IBM SPSS statistics Version 22.0, 2020).

## 3. Results

Data showed that a high percentage of carcasses (n = 503,000) resulted from random and unidentified crossbreeding (51.3%). The statistical data processing showed that the carcass weight and the age of slaughter averaged 298.9 ± 0.2 kg and 559.1 ± 0.3 days (about 1.5 years), respectively.

### 3.1. The Effect of Breed on Carcass Characteristics

The total number of carcasses in Table 1 was 321,381. The breeds with the largest number of carcasses were Limousin (28.8%) and Holstein (21.7%). Additionally, 12.9% of beef carcasses slaughtered were from the local breed of Greek Red. It is worth noting that a remarkable number of carcasses were Metis (9.5%) and Baltata Romameasca (6.7%), breeds originated mainly from Romania, a favorable destination to buy cattle for fattening due to its short distance from Greece.

Mean carcass weight ranged from 171.3 ± 2.3 kg (Vrachiceratiki) to 425.6 ± 3.1 kg (Parthenaise). Table 1 showed that an average carcass weight over 400 kg was observed for meat beef breeds as Parthenaise (425.6 ± 3.1 kg) and Blonde d’Aquitaine (404.9 ± 1.0 kg) and the crossed type Groase (405.4 ± 0.8 kg). Lower mean carcass weight was found in carcasses from local cattle breeds with small body conformation as Vrachiceratiki (171.3 ± 2.3 kg), Greek Buffalo (200.1 ± 1.1 kg) and Local (206.8 ± 1.2 kg). In contrast carcasses from Greek Red (251.5 ± 0.4 kg) and Greek Blonde (290.3 ± 3.4 kg) had higher mean carcass weight among the local breeds and good body conformation because the animals were upgraded crossbred with Limousin and Charolais respectively. On the other hand, a relatively low mean carcass weight had the carcass from Holsteins (251.1 ± 0.4 kg).

**Figure 1:**
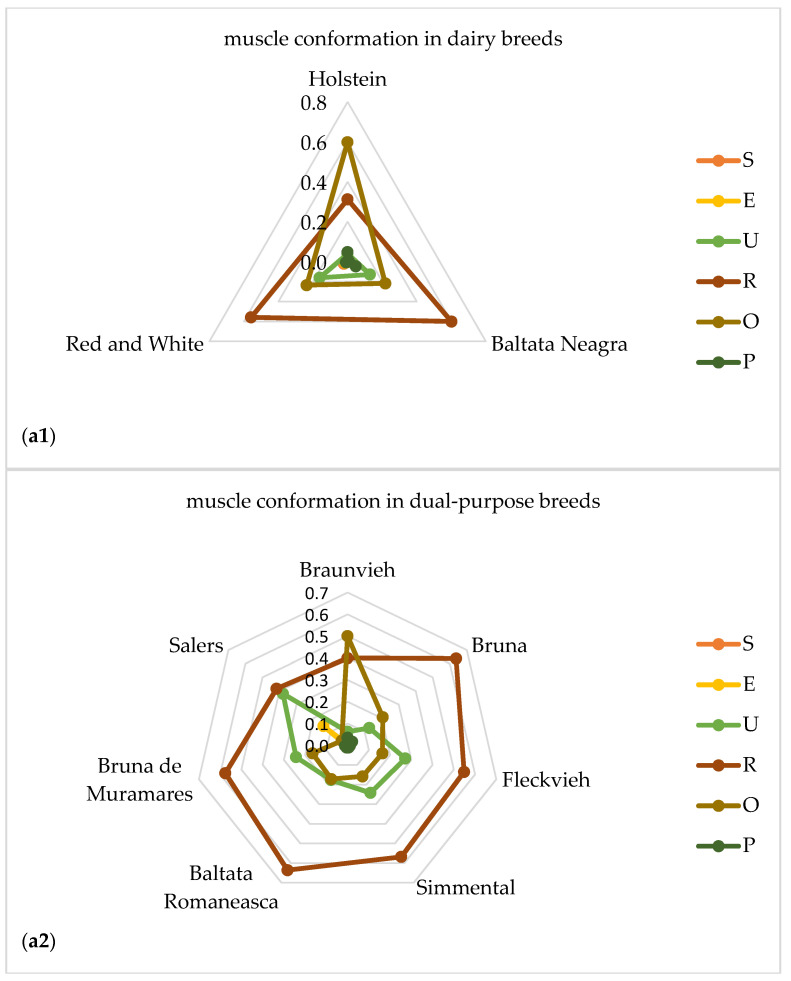

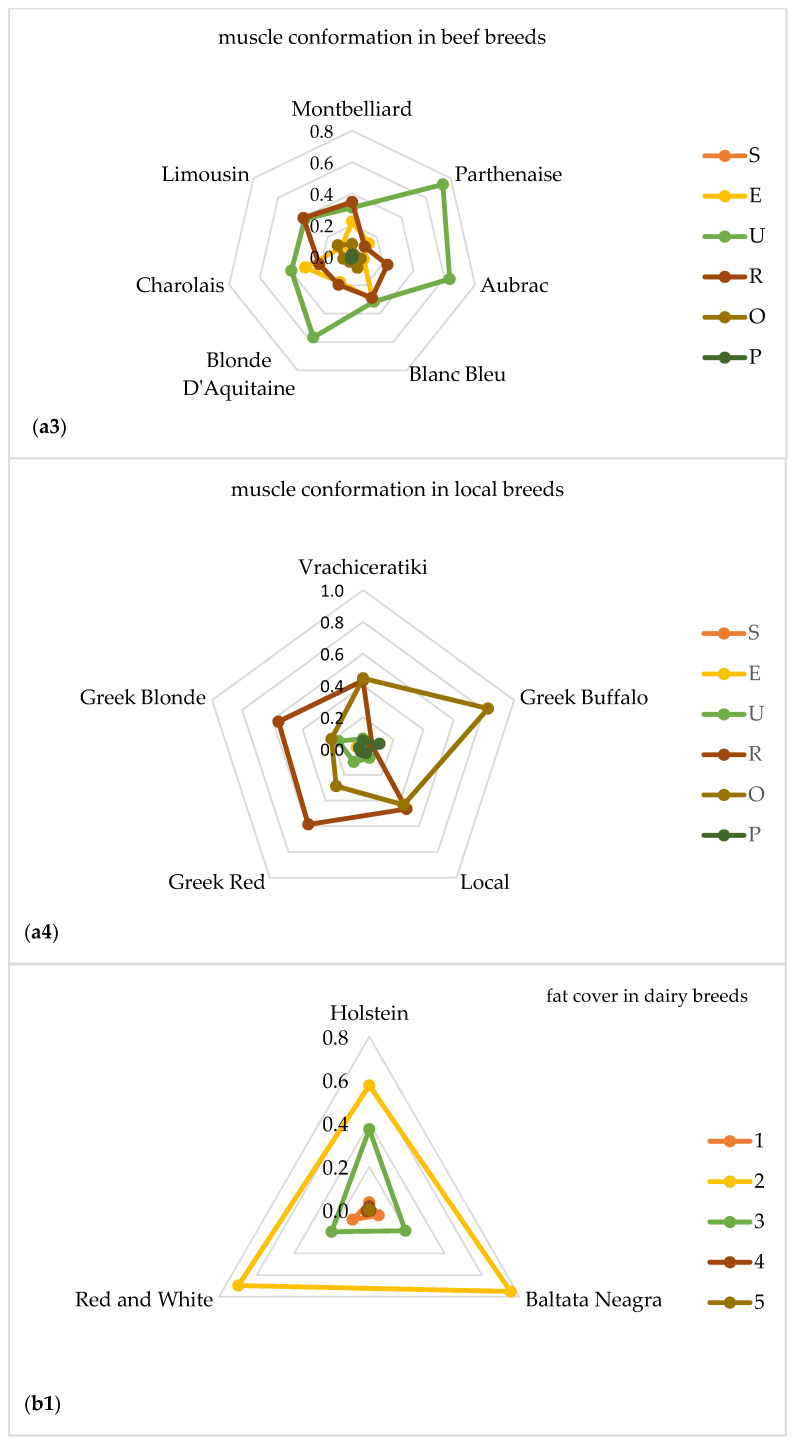

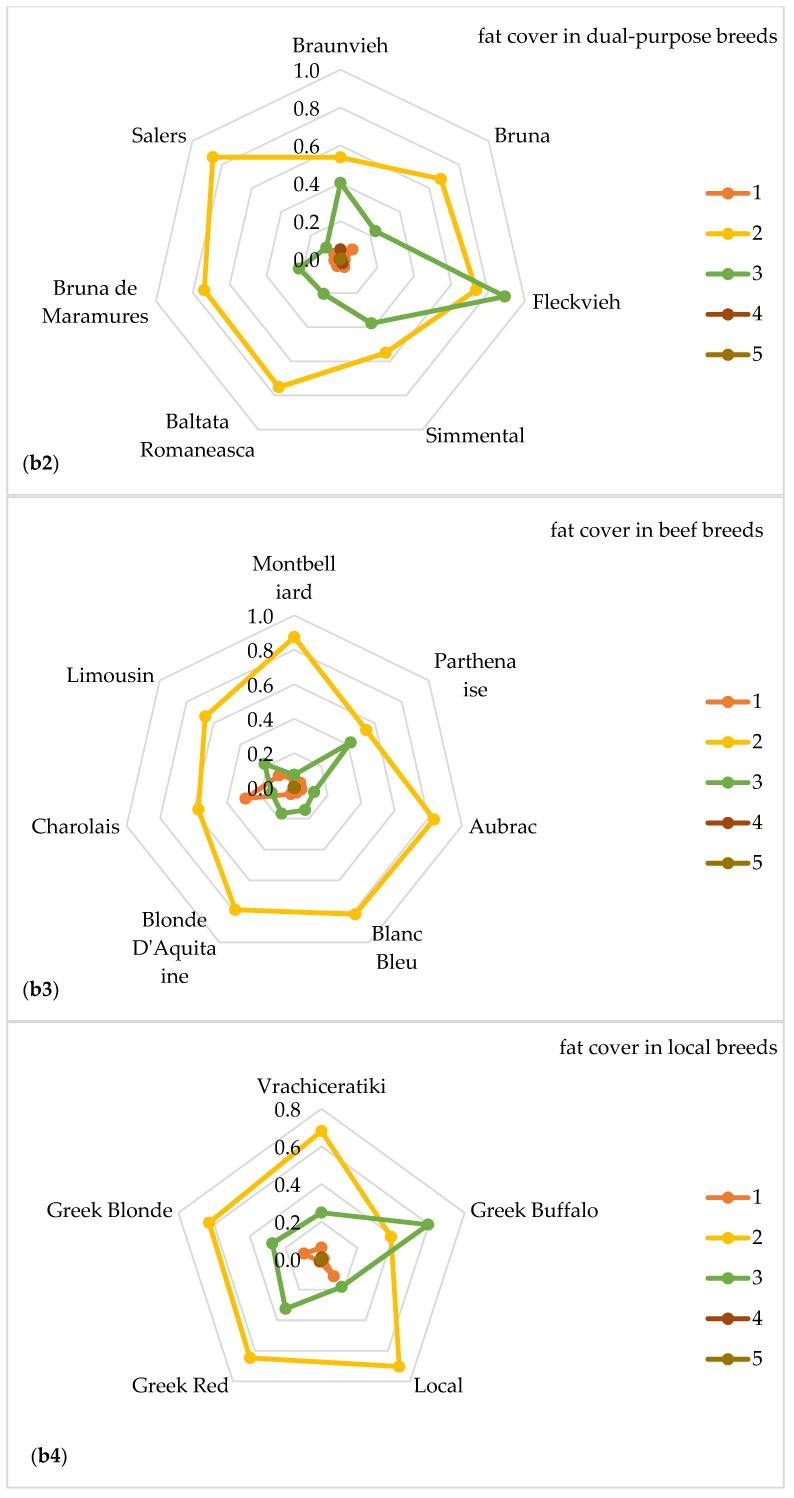
Proportions of muscle conformation (**a1**–**a4**) and fat cover (**b1**–**b4**) scores in grouped breed types of dairy (**a1**,**b1**), dual-purpose (**a2**,**b2**), beef (**a3**,**b3**) and local (**a4**,**b4**) cattle breeds according to SEUROP classification system.

Figure 1 shows the distribution of frequencies of classes for muscle conformation (a1–a4) and fat deposit (b1–b4) scores given as explanatory spider web charts in grouped breed types of dairy (a1,b1), dual purpose (a2,b2), beef (a3,b3) and local (a4,b4) cattle breeds, respectively.

According to the muscle conformation and the breed types of cattle it is obvious from the Figure 1 that the beef breeds had the highest value in conformation classes. The beef breeds that distinguished for their very good muscular conformation (class U) were Parthenaise, Blonde d’ Aquitaine and Charolais. From the dual-purpose breeds, only Salers had classified with SEUROP grid in class E. The local and the dairy breeds had similar muscle classification classes. Within class O, the classified carcasses were Holstein and Greek Buffalo. It is worth noting that for the fat deposit, the majority of carcasses in all breed types classified in class 2. The breeds that classified in class 3 were Holstein, Greek Buffalo, Parthenaise and Fleckvieh. Greek Buffalo carcasses, although they had the second lowest mean carcass weight (200.1 ± 1.1 kg) from all breed types, it was showed that they had higher fat deposit similar to Parthenaise (425.6 ± 3.1 kg) that had the heaviest mean carcass weight among all breed types.

Mean slaughter age more than 600 d was observed in carcasses of Greek Buffalo (694.4 ± 3.3 d), Montbelliard (620.3 ± 7.0 d) and Fleckvieh (604.2 ± 2.7 d). These were mainly breeds reared in semi-extensive and dual-purpose farms, suitable for milk and meat production or very resilient cattle, not only able to produce plenty of milk but also to withstand environmental difficult conditions. On the contrary, lower mean age of slaughter was found in carcasses of the Greek Red (471.9 ± 0.7 d).

### 3.2. The Effect of Gender on Carcass Characteristics

The carcass weight of male carcasses (n = 268,463) was found significantly heavier (316.0 ± 0.2 kg) than females’ (n = 54,583) which was observed to be 214.3 ± 0.3 kg (*p* ≤ 0.001). The mean age of slaughter for male animals was 564.4 ± 0.3 d, while for females was 532.8 ± 0.8 d (*p* ≤ 0.001). It is worth noting the fact that female carcasses of this study came from a large percentage of heifers, intended primarily, for slaughter and not for replacement of older females. As common reasons to remove females under 2 years of age from the breeding herd, referred the low daily gains before weaning, questionable inheritance, poor performance of dam and/or sire, undesirable conformation, or failure to exhibit a normal oestrus cycle [32]. The age of slaughter was shorter for females because they were destined to be bred only for fattening. Females slaughtered at older age and heavier carcass weight had increased fat composition rather than increased muscular conformation. The opposite effect would be for male carcasses.

### 3.3. The Effect Of Year Of Slaughter On Carcass Characteristics

**Figure 2:**
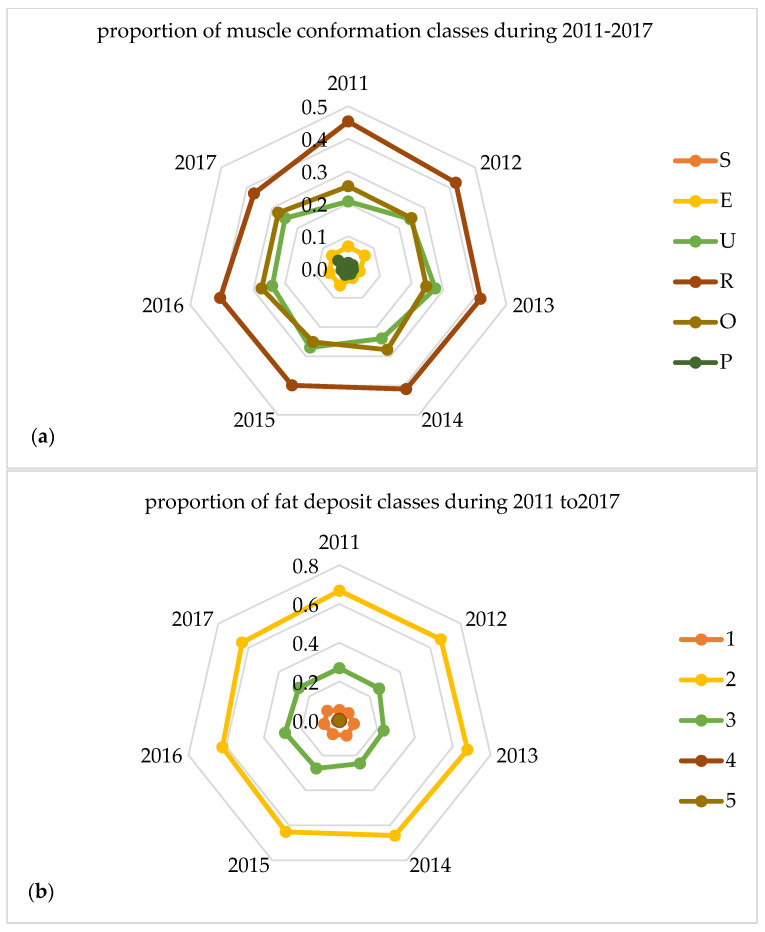
Proportion of muscle conformation (**a**) and fat deposit (**b**) classes for the carcasses during 2011–2017.

The year of slaughter affected the mean carcass weight and mean age of slaughter significantly (*p* ≤ 0.001) as it is shown in Table 2. Regarding the distribution of muscle conformation and fat deposit classes of carcasses during the seven years (2011–2017) as indicated by the SEUROP classification system the findings are presented in Figure 2, given as explanatory spider web charts. 

Over the course of seven years, fluctuations in both mean carcass weight and age of slaughter regardless of the gender were observed. The total number of cattle slaughtered was decreased over the years. In fact, the highest decrease was observed in 2016 and 2017, and reached the reduction rate of approximately 31.4% and 29.2%, respectively, compared to 2011, where it reached the highest value.

In Figure 2 the majority of carcasses were classified according to muscle conformation in classes R, O and U, while for the fat deposit in classes 2 and 3.

### 3.4. The Effect of Geographical Region of Farms on Carcass Characteristics

The largest number of cattle farms in the country was located in Northern Greece and specifically in the regions of Eastern Macedonia and Thrace and Central Macedonia and the lowest number was located in the region of Attica [33]. Table 3 shows that from the records of 322,609 cattle slaughtered in the 13 regions, the lightest carcass weight was found for carcasses derived from the region of Epirus (247.2 ± 0.8 kg). This observation is in accordance with the relatively small number of cattle farms in this area that cover only 7.5% of the total number of farms in the country. Taking into account the geographical criteria, the region of Epirus is a mountainous area in the northwestern part of the country where traditionally bred sheep and goats. Furthermore, the largest percentage of carcasses (32.2%) in the region of Epirus originated from Holstein breed, while 21.6% of them belonged to Greek breeds (Τable S1) that showed low mean carcass weight as mentioned in Table 1.

Regarding the mean age of slaughter, it is interesting to note that the lowest slaughter age observed in the region of Thessaly, could be attributed to the number of cattle raised for fattening in this region. The total percentage reached 16.74% of the total number of carcasses, as well as the fact that 39% of these carcasses originated from Limousin breed and 38.4% from the local breed Greek Red (Table S1).

Furthermore, in Table 3, we observed that only in three regions (Western Greece, Ionian Islands and North Aegean) occurred the highest value of the mean age of slaughter (over 600 d) and the mean carcass weight (over 300 kg). In addition, the cattle breed that slaughtered in these regions was mainly Limousin, a pure meat breed (data in Supplementary Table S1). 

**Figure 3:**
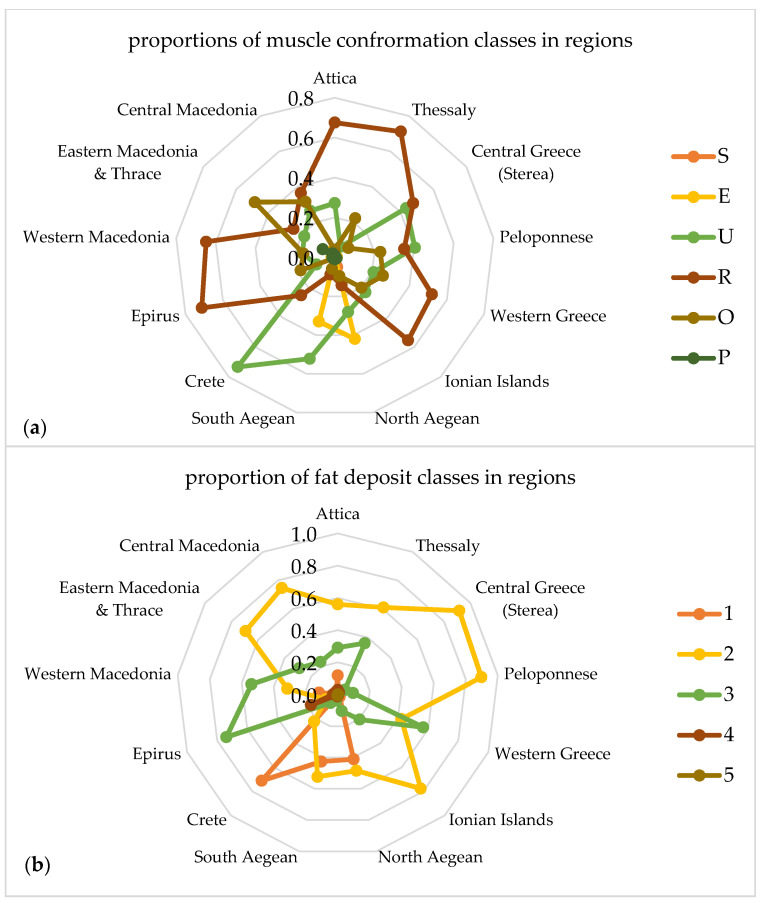
Proportions of muscle conformation (**a**) and fat deposit (**b**) classes distributed across 13 regions.

Figure 3 given as explanatory spider web charts depicts the proportion of muscle conformation (a) and fat deposit (b) classes in the 13 regions of the country.

As it shown in Figure 3, the major proposition of muscle conformation classes of the Greek carcasses in all over the 13 regions was R. In the region of Grete, a wide proportion of carcasses is classified in conformation class U and in the Eastern Macedonia & Thrace, a large proportion of carcasses classified in class O. The class E appeared mainly in carcasses slaughtered in the North Aegean. According to fat deposit the class 2 appeared in all 13 regions and only in regions of Epirus and Grete there was a large proportion that classified their carcasses in classes 3 and 1, respectively.

### 3.5. The Effect of Month of Slaughter on Carcass Characteristics

Regarding the total number of carcasses had significant differences between the first and the second half of the year (Table 4). It is worth noting that a remarkable number of carcasses (over 30,000) were slaughtered in the second half of the year especially on the 7th, 8th, 11th and 12th month. On the contrary the lowest number of carcasses showed on the 1st month of the year (n = 8997).

Comparisons among the months of slaughter showed that both the mean carcass weight and the mean age of slaughter differentiated significantly (*p* ≤ 0.001). The lowest value for the mean carcass weight was in the 3rd month of the year (293.2 ± 0.7 kg) while the highest value was in the 11th month (301.4 ± 0.6 kg). The difference between the two values of the mean carcass weight was 8.2 kg.

As for the age of slaughter the lower value appeared in 5th and 6th month of the year (555.3 ± 0.9 d and 555.4 ± 0.9 d respectively) and the highest in the 1st month (568.1 ± 1.6 d).

**Figure 4:**
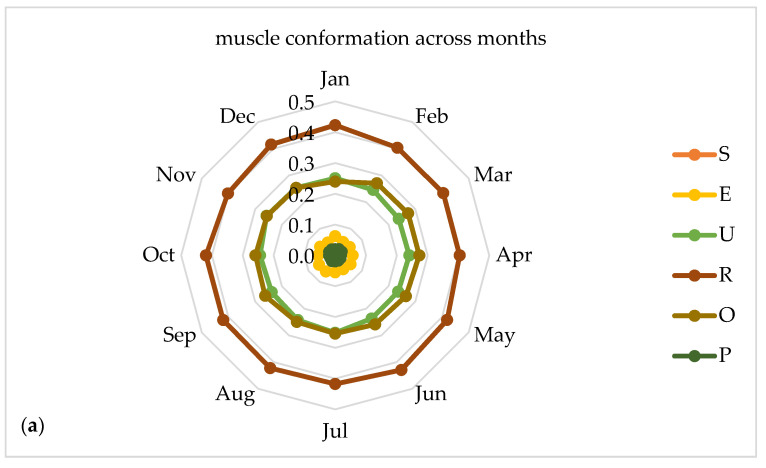

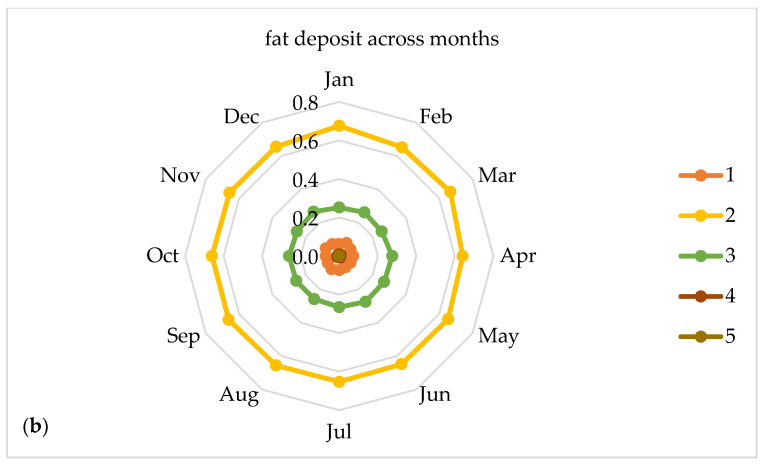
Proportions of muscle conformation (**a**) and fat deposit (**b**) classes distributed across 12 months.

In Figure 4 given as explanatory spider web charts, the distribution of conformation classes is homogeneous across the months and the highest proportion of muscle conformation in class R is widespread all over in the 12 months. Similarly, regarding the fat cover the highest proportion of fat deposit is classified in class 2.

### 3.6. Evaluation of Carcass Quality Characteristics Based on the SEUROP Classification System

Regarding the classification of carcasses into 6 categories (A,B,D,E,Z,V) based on gender and age at slaughter (Table 5), the heaviest mean carcass weight was recorded in the male carcasses of category B (329.2 ± 0.6 kg) and A (320.7 ± 0.2 kg).As for the female carcasses (categories D and E) the mean carcass weight ranged from 239.1 ± 0.8 kg and 219.4 ± 0.4 kg, respectively. Finally, in categories Z and V, it was appeared a lower mean carcass weight (218.1 ± 0.5 kg and 187.4 ± 1.6 kg, respectively) due to their younger age at slaughter.

According to Table 5 the largest percentage of carcasses (67.7%) belonged to category A (n = 218,732). The main reason was that the cattle imported to the country with the main purpose of breeding was fattening, they were preferred to be male, due to higher growth rate, better carcass performance and muscle conformation. Category V (animal carcasses under eight months old) had the lowest number of carcasses (n = 2,535) depicting the great need of Greek beef farmers to buy calves for breeding and fattening from abroad, as long as the demand was not met by the local market.

Regarding the classification of carcasses by category of muscle conformation according to SEUROP (Table 5), it is observed that the largest percentage of them were classified in class R (n = 130,302) as “good”, followed by the other classes (O, U, E, P & S). These findings are explained by the fact that the majority of carcasses were male animals that were mainly classified in category R. Additionally, in category P where the number of carcasses was relatively low (n = 6515), they were classified carcasses of poor muscle conformation. In addition, mean carcass weight followed by a normal distribution with the highest value appearing in category S (446.7 ± 1.8 kg) and the lowest in category P (209.4 ± 1.0 kg). The mean carcass weight of category R was 286.7 ± 0.2 kg. 

As for the mean age at slaughter, it is noticed that in category P there was a relatively extended number of days (600.6 ± 2.5 d) which could be explained by the fact that in this category mainly female carcasses were classified. Female cattle from this category removed from the livestock farm to the slaughterhouse due to low milk yields or possible accidents in the farm.

Regarding to the fattening state, most carcasses (n = 199,138) classified to class 2 followed by classes 3, 1, 4 and final class 5. The majority of carcasses in Greece (65.5%) had low fat deposit (class 1) while very large fat deposit (class 5) was found in a very small number of carcasses (n = 196).

## 4. Discussion

Our study based on a collection of seven-year records is a first attempt to give insight into the beef carcasses characteristics that produced in Greece and it will try to highlight trends that emerge into the European beef sector. Nearly the half of carcasses derived from crossbred animals of unknown genotype and this is a fact that arises from the need to supply Greek cattle farms with animals from other EU member states or from third countries, where the purchase of such crossbred animals is achieved at a lower price than that of purebred beef meat breeds. Additionally, many crossbred carcasses are offspring of dairy cows mated with bulls of meat breeds, which are also fattened in order to obtain the desired carcass weight.

Analyzing the purebred carcasses all investigated factors significantly affected the mean slaughter age and carcass weight. The mean carcass weight (298.9 ± 0.2 kg) compared with the average carcass weight in the EU-27 a decade ago, shows a trend to increase through this seven-year’s period [34]. According to the above source [34], the average carcass weight has increased continuously since 2002 in the EU. In comparison with the beef sector in Ireland [35], the average carcass weight that failed to achieve a desired conformation score, was 301 kg; hence, huge prospect exists to improve this parameter in Greek carcasses too. The explanation could be the same for Irish beef carcasses. The carcasses that failed to achieve the desired fat or weight specification, on the one hand, could be attributed to the inability of producers to determine whether an animal is suitable for slaughter and on the other hand, could be the inability of cattle to reach a desirable carcass [35].

In our study, the mean age at slaughter of females was 532.8 ± 0.8 d, because the older female carcasses usually classified in higher age of slaughter, were excluded from our analysis. The observations in this study were consistent with previous results about the effect of breed on carcass characteristics. In a study considering 15 European cattle breeds [36], the dairy and local cattle breeds produced lighter carcasses as opposed to predominantly cattle breeds. Breed-specific differences in growth rate of local breeds could explain their relatively lower carcass weights as it was pointed out in another study [37]. Similarly, in our study lower mean carcass weight was found mainly in carcasses of local cattle breeds with moderate to poor body conformation. Additionally, we found that higher value of slaughter age generated heavier carcass weight which is in line with the observations of [38]. Regarding the use of dairy Holstein calves for beef production it is a common practice, which represents a significant portion of the meat consumed worldwide. As it is mentioned [39], Holstein calves finished in feedlot had higher fat content in carcass than those finished on pasture and they are excellent producers of lean meat, with good smoothness, flavor and juiciness. In this study, the descendants of Holstein dairy cows were sold to light live weight because it was not economically advantageous to be fattened into heavy ones.

The breed type was also reflected in muscle conformation and fat cover classes. The highest conformation classes such as E and U, was found in the beef meat breeds and the lowest classes such as O and P in dairy breeds. These results are in line with the results of [36] where the highest conformation score was in the double muscled Piedmontese and the lowest in Jersey. On the contrary, all the breed types in our study for the fatness classes were ranged in a similar way, mainly in the class 2. Similar results have reported in [40] that bulls showed greater muscle development, less fat deposition and were more efficient in producing leaner carcasses than steers which may be mainly attributed to the effects of male hormones on muscle protein anabolism. The class E in muscular conformation, which classified Salers carcasses in our study could explained according to the results of [41] where between Holstein and Salers breeds were observed that Salers cows had more muscle in carcass and Holstein cows were fatter than Salers cows.

Furthermore, the gender as a factor had a significant influence on mean carcass weight and on mean age at slaughter (*p* ≤ 0.001). Similar to our findings it is referred by other studies [42,43] that bull carcasses are characterized by higher meat content with simultaneous lower content of fat compared to heifer carcasses. In addition, in a study with the double-muscled Belgian Blue bulls and cows, most of the carcass quality parameters were more favorable for males than for females [44]. In heifer’s life, rearing factors applied during both pre-weaning and fattening periods influenced carcass and meat quality [45]. The relationship between tenderness and gender has evaluated by many studies [40,46] which found that meat from young bulls was significantly less tender than that of heifers. The male carcasses in this study came almost exclusively from cattle, which, whether imported from other countries or born in the country and they were bred for fattening and slaughtered when they gained the desired live weight. Considering that the local market system is based on carcass weight, the heaviest young bulls have a significant economic advantage over heifers in commercial scale. Therefore, this result explains further the dominance of young bulls in the local slaughter of beef meat. In addition, the encouraged to produce heavier carcasses due to favor slaughter pricing of heavier carcasses is a common practice in many countries according to a study in South Africa [47].

The effect of year of slaughter on carcass traits reflects to a large extent the fluctuations of environmental factors on the cattle farms from year to year and the ability of beef industry to adapt and respond. In a study of Slovenian cattle [48], the carcass weight of young bulls, heifers and cows varied among different years, but no trend could be noticed. Additionally, in Slovenia, in another study [49] within a decade from January 2005 to December 2015, the carcass weight of young bulls significantly increased from 345 to 354 kg in the first three years and then to 359 kg in 2013. The decline in the total number of beef carcasses was due to the outbreak of bovine nodular dermatitis in the country during 2016–2017 which affected mainly the areas of Northern Greece where the largest number of cattle farms exist. In addition, the decline trend for the number of male carcasses reaching 32.88% from 2011 to 2017, could be attributed to the same reason mentioned for the total decreased number of carcasses, since male carcasses made up a 83% of the total number of cattle slaughtered in the country.

Comparisons of the classification categories within the geographical regions of the cattle farms, it was observed that the best performance in terms of the carcass muscle conformation, number of carcasses and breed types was located in the Northern and Central regions of Greece. There is an obvious heterogeneity of the environment affecting the productive management of cattle farms in our country. Similar to our findings, cattle carcasses from the northern regions of Mexico had a higher marbling score than those in the southern regions and performed better overall [50]. The carcasses classification according to muscle conformation, focusing in the region of Central Macedonia showed that 37% of the total carcasses were slaughtered in the above region. Within this region a percentage of 26.4% of the total number of carcasses were classified in category U, 3.51% in category E and 36.38% in category R, while only 2.24% in category P. In addition to that, in the region of Central Macedonia, the mostly high-yielding cattle breeds were reared. More specifically, 75.25% of the total carcasses of the Blanc Blue breed and 77.3% of the total carcasses of the Blonde d’ Aquitaine breed were bred and slaughtered in this region (Τable S1). It is reasonable to consider that the cattle farmers of the above region seem to be more professionals regarding the management of their livestock and presented a business profile that focuses on their economic performance.

Significant differences were found among months of slaughter (*p* ≤ 0.001). According to several studies on this factor, the seasonal changes in temperature affect the level of glycogen after slaughter and the ultimate pH and therefore the quality of meat [51,52]. In addition, another study [53] showed that the quality classification grades of the carcass were higher during January, February and March compared with May through November. The above results [53] are in line with ours. It is worth noting that in another study [54] the annual trends typically reach the lightest Hot Carcass Weight (HCW) for the year in May and seasonal differences in HCW could be a result of the type of cattle marketed at this period. Hence, similarly, in our study the low mean carcass weight in March could be due to a lesser availability of high nutrition value feeds during late autumn and winter seasons or market issues. Furthermore, our results were consistent with the results of [38], that heavier carcasses were observed for slaughter in autumn and winter. These results confirmed by another study [55], where animals that slaughtered in spring recorded lower carcass weights. It is known that cattle imported for fattening during the summer months are slaughtered during the winter. The average fattening period is about five months. Summer season, due to the extreme weather conditions (high temperature, high humidity) prevailed stresses and disrupts the growth rate of the animals. As it is known [56], cattle are considered more sensitive to hot than to cool temperatures. As a result, those cattle have not gained sufficient live weight. On the contrary, in December the largest number of carcasses (n = 32,567) was observed due to the efforts to satisfy the high demand during the Christmas period, while the mean carcass weight was increased (300.1 ± 0.6 kg). Hence, the lowest number of carcasses slaughtered in the first month of the year reflects the decrease of meat consumption after holiday’s period. It is also worth noting that the mean age at slaughter over 562 days was higher mainly during the winter months, from November to February, to allow animals acquire the desired carcass weight to cover the high consumption observed during this period.

The EU classification system presents differences on beef carcass quality among the member states. The variations in cattle delivered to a European slaughterhouse in terms of age, breed, weight and feeding production systems are large and make it very difficult or even impossible for the slaughtering industry to produce European beef of a standardized quality [57]. Hence, the comparison between beef carcasses that classified under SEUROP classification system could lead to useful information about the beef sector in EU. The results in the present study showed that the majority of carcasses were classified in the category R and in class 2 of fat cover, i.e., carcasses with good muscle conformation and low amount of fat. Fat cover is a more reliable indicator of meat quality than carcass conformation [58]. On the other hand, carcass conformation classes are a factor that influences purchase prices. It is notable that in another study in Poland in all cattle categories, the better the conformation class, the higher the purchase price [59]. In accordance with that the Spanish beef market demands young bullock cattle with superior muscling that will yield a higher percentage of lean, and therefore, carcass conformation is the key factor for carcass economic value [60]. The results of the latter study for low fat carcasses are in accordance with a study conducted in Finland [61] where consumers favor in low fat products. The above studies have motivated beef industry to suggest that two thirds of the carcasses would have a EUROP fat score of 2 and one third a EUROP fat score of 3 and also to give penalties for carcasses less than 320 kg with fat scores 3–5 and for carcasses over 320 kg with fat scores 4–5. In France [18] although conformation has been a more important component, French consumers prefer beef with less visual fat at the retail level. In contrast, this trend for low fat carcasses if it is compared with other European but not EU member states, the results did not converge. For example, in Serbia [62], beef carcasses were evaluated as having conformation R in 59% of cases but the carcass fat tissue coverage degree was rated as 4 for 87% of carcasses.

## 5. Conclusions

In Greece, the carcasses are produced from a variety of cattle breed types. In our study, beef breeds classified in highest muscle conformation classes such as E and U, while in lowest classes such as O and P classified mainly dairy and local cattle breeds. From the dual-purpose breeds only Salers had a large proportion of classified carcasses in class E. Local breeds and Holstein cattle had lower mean carcass weight and in comparison with other EU countries, the lower value of the mean carcass weight in main beef breeds that produced in Greece it is due to different breeding and diet conditions. Mean carcass weight and mean age at slaughter were significantly differed among the relative levels of each factor examined. Male carcasses were 83% of the total number of cattle slaughtered in Greece, which reflects the dominance of young bulls in the local market system. There was a decreasing trend in the total number of cattle reared for meat during the studied years. Northern and central regions of Greece produced carcasses with the best performance in terms of carcass muscle conformation, number of carcasses and breed types, so the development of beef sector in Greece is based mainly on these regions. Higher values of mean carcass weight and mean age at slaughter were observed in winter than in the summer months. According to the SEUROP classification system, Greek carcasses had good muscle conformation (class R) and low amount of fat (class 2), which could reveal an EU trend for low fat deposit in beef meat.

## Figures and Tables

**Table 1:** Number of carcasses per breed (N), means ± std. error for the age at slaughter (days, d) and the carcass weight (kg) from 24 cattle breeds (>100 observations) reared in Greece.

Breed Type	Breed Name	N	Age at Slaughter (d)	Carcass Weight (kg)
dairy	Holstein	69,861	578.0 ^cghk^ ± 0.6	251.1 ^c^ ± 0.3
dairy	Red and White	596	529.1 ^ad^ ± 6.6	269.0 ^gko^ ± 4.6
dairy	Baltata Neagra	562	572.6 ^bdefg^ ± 7.4	274.2 ^dglp^ ± 3.5
dual	Braunvieh	1952	582.7 ^cfh^ ± 3.4	262.1 ^kl^ ± 1.7
dual	Bruna	683	574.1 ^behijl^ ± 6.2	275.3 ^dlno^ ± 2.8
dual	Fleckvieh	2381	604.2 ^ck^ ± 2.7	292.3 ^pr^ ± 1.6
dual	Simmental	6778	591.6 ^cjk^ ± 1.6	293.3 ^pt^ ± 1.1
dual	Baltata Romameasca	21,461	566.2 ^bel^± 1.0	303.7 ^fh^ ± 0.5
dual	Bruna de Maramures	801	565.0 ^bdeh^ ± 5.4	304.8 ^dhrt^ ± 3.0
dual	Salers	530	581.3 ^behkl^ ± 5.1	360.4 ^jq^ ± 3.2
beef	Limousin	92,560	568.3 ^b^ ± 0.4	328.9 ^s^ ± 0.3
beef	Montbelliard	513	620.3 ^c^ ± 7.0	343.2 ^q^ ± 3.7
beef	Aubrac	6851	565.3 ^bel^ ± 1.3	373.6 ^ejnr^ ± 1.1
beef	Blanc Bleu	1701	574.2 ^behl^± 2.8	378.9 ^e^ ± 2.3
beef	Charolais	13,326	599.6 ^ck^± 1.1	388.7 ± 0.8
beef	Blonde d’ Aquitaine	7898	528.0 ^a^ ± 1.1	404.9 ^i^ ± 1.0
beef	Parthenaise	743	593.3 ^cgjk^ ± 3.6	425.6 ^m^ ± 3.1
crossed	Metis	30,517	563.8 ^dl^ ± 0.9	293.4 ^dp^ ± 0.5
crossed	Groase	8993	554.4 ^i^ ± 1.1	405.4 ^i^ ± 0.8
local	Vrachiceratiki	1488	528.1 ^ad^± 4.6	171.3 ^b^± 2.3
local	Greek Buffalo	2493	694.4 ± 3.3	200.1 ^a^ ± 1.1
local	Local	6344	567.5 ^cd^± 2.1	206.8 ^a^ ± 1.2
local	Greek Red	41,358	471.9 ^c^ ± 0.7	251.5 ^c^ ± 0.4
local	Greek Blonde	991	547.2 ^di^ ± 4.5	290.3 ^dp^ ±3.4

Means within the same column followed by different superscript for each variable (^a, b, c, d, e, f, g, h, i, j, k, l, m, n, o, p, q, r, s, t^) among breeds differ significantly *p* ≤ 0.05.

**Table 2:** Number of carcasses, means ± std. error for the age of slaughter (days, d) and carcass weight (kg) during 2011 to 2017.

Year	N	Age of Slaughter (d)	Carcass Weight (kg)
2011	58,652	552.5 ^a^ ± 0.6	294.8 ^a^ ± 0.4
2012	52,634	560.4 ^b^ ± 0.7	296.2 ^a^ ± 0.4
2013	45,887	565.0 ^c^ ± 0.7	299.7 ^b^ ± 0.5
2014	42,514	563.6 ^c^ ± 0.7	299.5 ^b^ ± 0.5
2015	41,625	564.2 ^c^ ± 0.7	305.3 ^c^ ± 0.5
2016	40,229	557.5 ^b^ ± 0.8	303.5 ^c^ ± 0.5
2017	41,505	552.1 ^a^ ± 0.7	295.2 ^a^ ± 0.5
total	323,046	559.1 ± 0.3	298.8 ± 0.2

Means within the same column followed by different superscript for each variable (^a, b, c^) among years differ significantly (*p* ≤ 0.05).

**Table 3:** Number of cattle farms and beef carcasses (Ν), means ± std. error for the age at slaughter (days) and carcass weight (kg) distributed in the 13 geographical regions.

Geographical Region	N (Farms)	N (Carcasses)	Age at Slaughter (d)	Carcass Weight (kg)
ATTICA				
1. Attica	53	3684	522.2 ^a^ ± 3.2	268.2 ^a^ ± 1.3
CENTRAL GREECE				
2. Thessaly	2	53,994	498.5 ^b^ ± 0.6	270.0 ^a^ ± 0.4
3. Central Greece (Sterea)	573	15,578	527.1 ^a^ ± 1.1	297.7 ^b^ ± 0.8
4. Peloponnese	920	8914	569.5 ^c^ ± 1.5	268.0 ^a^ ± 1.3
5. Western Greece	1591	26,924	608.3 ^di^ ± 0.9	369.9 ^e^ ± 0.7
6. Ionian Islands	274	4152	623.0 ^ei^± 2.1	344.4 ^f^ ± 1.5
AEGEAN ISLANDS & CRETE				
7. North Aegean	551	9200	616.3 ^f^± 1.4	316.6 ^g^ ± 0.9
8. South Aegean	1183	7035	567.0 ^dg^ ± 1.8	279.0 ^dh^ ± 1.0
9. Crete	200	8459	572.0 ^h^ ± 1.7	259.8 ^i^ ± 0.9
NORTHERN GREECE				
10. Epirus	1103	12,058	544.8 ^dei^ ± 1.5	247.2 ^c^ ± 0.8
11. Western Macedonia	1099	14,185	561.5 ^e^ ± 1.2	275.5 ^d^ ± 0.8
12. Eastern Macedonia & Thrace	2946	39,078	565.3 ^i^ ± 0.8	268.9 ^a^ ±0,4
13. Central Macedonia	2642	119,348	570.6 ^e^ ± 0.4	318.0 ^gj^ ± 0.3
total	14,699	322,609	559.0 ± 0.3	298.8 ± 0.2

Means within the same column followed by different superscript for each variable (^a, b, c, d, e, f, g, h, i, j^) among geographical regions differ significantly (*p* ≤ 0.05).

**Table 4:** Number of beef carcasses per month, means ± std. error for the age at slaughter (days, d) and carcass weight (kg).

Month of Slaughter	N	Age at Slaughter (d)	Carcass Weight (kg)
1	8997	568.1 ^a^ ± 1.6	302.7 ^a^ ± 1.1
2	25,758	564.6 ^ad^ ± 0.9	298.5 ^bde^ ± 0.6
3	20,951	557.2 ^b^ ± 1.1	293.2 ^c^ ± 0.7
4	25,492	561.9 ^cde^ ± 0.9	298.6 ^bde^ ± 0.6
5	28,682	555.3 ^b^ ± 0.9	298.3 ^bde^ ± 0.6
6	27,820	555.4 ^b^ ± 0.9	297.3 ^d^ ± 0.6
7	30,423	554.8 ^b^ ± 0.8	297.1 ^df^ ± 0.6
8	30,280	558.0 ^be^ ± 0.8	299.4 ^abd^ ± 0.5
9	29,495	557.4 ^b^ ± 0.8	300.7 ^ae^ ± 0.6
10	29,447	557.0 ^b^ ± 0.8	299.3 ^abd^ ± 0.6
11	30,879	563.1 ^ad^ ± 0.8	301.4 ^a^ ± 0.6
12	32,567	561.8 ^cde^ ± 0.8	300.1 ^ab^ ± 0.6
total	320,791	559.0 ± 0.3	298.8 ± 0.2

Means within the same column followed by different superscript for each variable (^a, b, c, d, e,f^) among month of slaughter differ significantly (*p* ≤ 0.05).

**Table 5:** Number of beef carcasses (N), means ± std. error for the age at slaughter (days, d) and the carcass weight (kg) according to SEUROP classification scale ^1^ (category, muscle conformation and fat deposit).

SEUROP Classification Scale	N	Age at Slaughter (d)	Carcass Weight(kg)
category *			
A	218,732	549.5 ^a^ ± 0.2	320.7 ^a^ ± 0.2
B	31,506	813.3 ^b^ ± 0.4	329.2 ^b^ ± 0.6
D	7597	835.4 ^c^ ± 1.1	239.1 ^c^ ± 0.8
E	35,465	540.0 ^d^ ± 0.7	219.4 ^d^ ± 0.4
Z	27,107	318.2 ^e^ ± 0.2	218.1 ^d^ ± 0.5
V	2535	226.2 ^f^ ± 0.2	187.4 ^e^ ± 1.6
muscle conformation **			
S	1389	730.5 ^a^ ± 3.8	446.7 ^a^ ± 1.8
E	17,360	590.7 ^b^ ± 0.8	422.2 ^b^ ± 0.5
U	76,802	580.3 ^c^ ± 0.4	372.6 ^c^ ± 0.3
R	130,302	546.8 ^d^ ± 0.4	286.7 ^d^ ± 0.2
O	81,670	561.0 ^e^ ± 0.6	235.0 ^e^ ± 0.3
P	6515	600.6 ^f^ ± 2.5	209.4 ^f^ ± 1.0
fat deposit***			
1	21,911	602.2 ^a^ ± 1.0	348.8 ^a^ ± 0.8
2	199,138	553.6 ^b^ ± 0.3	301.8 ^b^ ± 0.2
3	79,909	574.6 ^c^ ± 0.5	289.7 ^c^ ± 0.3
4	2647	583.8 ^d^ ± 3.7	257.7 ^d^ ± 1.8
5	196	579.2 ^abcd^ ± 11.0	273.3 ^cd^ ± 5.9

^1^ each class/category includes subclasses (+) & (−); *A: carcasses of uncastrated male animals aged from 12 months to less than 24 months; B: carcasses of uncastrated male animals aged from 24 months; D: carcasses of female animals that have calved; E: carcasses of other female animals aged from 12 months; Z: carcasses of animals aged from 8 months to less than 12 months; V: carcasses of animals aged less than 8 months; ** S: superior; E: excellent; U: very good; R: good; O: fair; P: poor; *** class 1 = low; class 2 = slight; class 3 = average; class 4 = high; class 5 = very high; means within the same column followed by different superscript for each variable (a,b,c,d,e,f) among; classes/categories differ significantly (*p* ≤ 0.05).

## Supplementary Materials

The following are available online at https://www.mdpi.com/2304-8158/9/12/1764/s1, Table S1: Number of carcasses (counts) classified for their muscle conformation in each breed and region and the relative percentages.
